# Performance of AAV8 vectors expressing human factor IX from a hepatic-selective promoter following intravenous injection into rats

**DOI:** 10.1186/1479-0556-6-9

**Published:** 2008-03-03

**Authors:** Tracey Graham, Jenny McIntosh, Lorraine M Work, Amit Nathwani, Andrew H Baker

**Affiliations:** 1 British Heart Foundation Glasgow Cardiovascular Research Centre, University of Glasgow, Glasgow G12 8TA, UK; 2 Department of Haematology, University College London, London, UK

## Abstract

**Background:**

Vectors based on adeno-associated virus-8 (AAV8) have shown efficiency and efficacy for liver-directed gene therapy protocols following intravascular injection, particularly in relation to haemophilia gene therapy. AAV8 has also been proposed for gene therapy targeted at skeletal and cardiac muscle, again via intravascular injection. It is important to assess vector targeting at the level of virion accumulation and transgene expression in multiple species to ascertain potential issues relating to species variation in infectivity profiles.

**Methods:**

We used AAV8 vectors expressing human factor IX (FIX) from the liver-specific LP-1 promoter and administered this virus via the intravascular route of injection into 12 week old Wistar Kyoto rats. We assessed FIX levels in serum by ELISA and transgene expression at sacrifice by immunohistochemistry using anti-FIX antibodies. Vector DNA levels in organs we determined by real time PCR.

**Results:**

Administration of 1 × 10^11 ^or 5 × 10^11 ^scAAV8-LP1-hFIX vector particles/rat resulted in efficient production of physiological hFIX levels, respectively in blood assessed 4 weeks post-injection. This was maintained for the 4 month duration of the study. At 4 months we observed liver persistence of vector with minimal non-hepatic distribution.

**Conclusion:**

Our results demonstrate that AAV8 is a robust vector for delivering therapeutic genes into rat liver following intravascular injection.

## Background

Adeno-associated viruses (AAV) are attractive vectors for *in vivo *gene therapy due to their safety profile and ability to achieve long term production of therapeutic genes following cell infection. Intravascular delivery provides a minimally invasive yet versatile approach to gene therapy for applications to correct liver deficiencies or utilise this organ for production of therapeutic genes. AAV serotype 2 (AAV2) has been studied in detail preclinically with success [1-5], however, this vector generates a cytotoxic T-cell response as evidenced in human subjects on a haemophilia trial leading to transient transaminitis and only short term FIX production [6]. Alternate AAV vectors are being pursued for gene therapy to overcome limitations in the efficiency of AAV2-mediated gene delivery to the liver [7]. AAV8 in particular shows promise in this regard since it is less immunogenic than AAV-2 capsids [8]. AAV8 has been shown to have improved liver transduction efficiency in murine models of 10–100 fold increase over AAV2 vectors [9] and rapid uncoating post-internalisation overcomes additional limitations of AAV2 [10]. A number of studies have already assessed the potential of AAV8 using mouse and non-human primate models for haemophilia gene therapy and application to muscular dystrophy [11-14]. Importantly, it has been demonstrated that peripheral venous administration is as effective as the direct intraportal route of vector administration in animal models suggesting profound liver targeting capacity [13]. In addition, Wang et al demonstrated the long-term efficacy and safety of the AAV2/8 vector in liver-directed gene therapy in dogs [15]. The application of the vector to gene therapy is therefore dictated by the tropism observed following administration by a particular route of administration. Subtle differences between species can have a large impact on efficiency and safety. No studies to date have addressed AAV2/8 biodistribution or transgene expression following *iv *delivery in rats, a particularly important model for many pre-clinical diseases. This study therefore sought to quantify both parameters following peripheral vein injection into adult male Wistar Kyoto rats. We used an AAV8 pseudotyped self-complementary AAV vector, with the liver-specific enhancer/promoter LP1 driving expression of the reporter gene hFIX [11,12] and assessed biodistribution, transgene expression and longevity of expression.

## Methods

### Animals

Animal procedures were performed in accordance with UK Home Office Animals (Scientific) Procedure Act 1986, UK and Personal Licences regulations. scAAV8-LP1-hFIX vector at doses ranging from 10^10^vg/rat to 5 × 10^11^vg/rat were administered to 12 week old Wistar Kyoto rats (n = 3/group) via femoral vein injection.

### Vectors

The construction and production of this vector was described previously [12]. Briefly, scAAV8-LP1-hFIX was constructed using a recombinant AAV2 vector backbone incorporating an expression cassette with the human factor IX gene and driven by a liver-specific enhancer/promoter LP1, and subsequently cross-packaged as a dimer into the AAV8 capsid protein. The scAAV8 pseudotyped vector was produced by the triple transient transfection method and purified by ion exchange chromatography. Vector genome titres were determined by the previously described quantitative slot-blot method using supercoiled plasmid DNA as standards [16].

### Quantitative analysis of vector DNA from tissues of scAAV8-LP1-hFIX infused rats to determine biodistribution profile of rAAV8 vector

Four months after vector infusions animals were sacrificed and tissues immediately snap frozen in liquid nitrogen then stored at -70°C. DNA concentrations were quantified by spectrophotometry and 100 ng of purified DNA from each tissue sample obtained was used in a Sybr Green PCR assay, with 200 nmol/L of sense (5'-TTTCCTGATGTGGACTATGT-3') and 200 nmol/L of anti-sense primer (5'-TCATGGAAGCCAGCACAGAACATG-3'), to quantify vector DNA by TaqMan PCR (Perkin-Elmer, Applied Biosystems, Foster City, California). A human FIX quantification standard curve was generated from 10-fold serial dilutions of scAAV8-LP1-hFIX preparation. Target template serial dilutions and all experimental samples were run in duplicate. The following reaction conditions were used: denaturation, 95°C for 10 minutes; amplification, 95°C for 15s, 60°C for 1 min (40 cycles); dissociation, 95°C for 15s, 60°C for 15s, 95°C for 15s using a 7900HT ABI Prism Sequence Detection System. Data were processed by the SDS 2.1 software package (Perkin-Elmer, Applied Biosystems).

### Determination of circulating human factor IX levels in blood plasma of scAAV8-LP1-hFIX infused rats

Blood samples were collected at days 0, 3, 7, 14, 28, 56, 112 days post injection into citrate buffer (9 parts blood: 1 part anti-coagulant) from the saphenous vein by venipuncture, starting prior to vector administration to obtain baseline measurements, throughout the study and then a final bleed by cardiac puncture at 4 months when rats were sacrificed. Citrate buffered blood samples were centrifuged at 2000 g for 15 minutes then the plasma was collected and subsequently assayed by anti-FX ELISA [12] to measure circulating hFIX activity.

### Histological analysis of human FIX expression in livers of scAAV8-LP1-hFIX infused rats

At 4 months after vector infusions pieces of rat liver lobes were fixed for 16 hours in 10% formalin. Formalin fixed, paraffin embedded specimens were sectioned at 6 μm using a microtome (Shandon Finesse 325, Thermo Electron Co.). Heart tissues were also collected and processed in the same way and used as negative controls for human FIX expression. Sections were deparaffinised and rehydrated in an alcohol concentration gradient then endogenous peroxidase activity was quenched by incubating the sections in 0.3% hydrogen peroxide in methanol for 30 minutes. Unmasking of the antigen was performed using Target Retrieval Solution as per the recommendations of the manufacturer (Dako Cytomation Ltd, UK) then sections were blocked in PBS for 30 minutes before proceeding with staining.

Human FIX expression in tissue sections was analysed by immunohistochemistry using a 1:100 dilution (10 μg/ml) of goat anti-human FIX-HRP antibody (Affinity Biologicals). The negative control was goat IgG used at 10 μg/ml and anti-goat IgG HRP conjugated antibody at 1:10000 (Dako Cytomation Ltd, UK). The primary antibody was incubated overnight at 4°C and the secondary antibody was incubated at room temperature for 1 hour. Diaminobenzidine (DAB) chromagen solution (Dako Cytomation Ltd, UK) was used to detect horse radish peroxidase activity (positive staining shows as brown) and cell nuclei were counterstained in haematoxylin. Sections were dehydrated through an ethanol gradient and mounted in Histomount (Invitrogen). Images were captured by a Hitachi HVC2OA camera attached to an Olympus ×40 microscope.

## Results and Discussion

**Figure 1:**
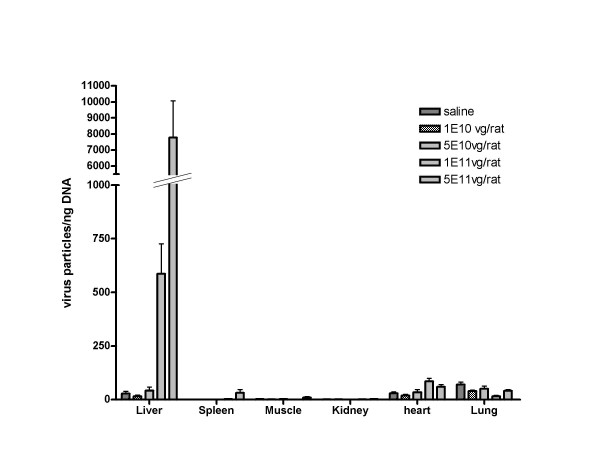
**Quantitative analysis of vector biodistribution following intravascular administration of AAV8LP-1FIX into rats**. DNA was extracted and processed from tissues of rats, which were dissected 4 months after intravenous injections of scAAV8-LP1-hFIX. Purified DNA was subsequently analysed by quantitative real-time PCR using hFIX specific primers designed to hybridise to the human FIX coding region of the AAV8 vector.^12^. Data are presented as the means ± standard error of the mean. VG = vector genomes.

We examined the transduction efficiency and biodistribution profile of scAAV8LP1hFIX in 12 week old male Wister Kyoto rats 4 months following a single systemic injection of the vector via the femoral vein at doses of between 10^10^vg/rat to 5 × 10^11^vg/rat or saline control (n = 3 rats per group). The tissue distribution (measured by Taqman™ realtime PCR) of the scAAV8-LP1-hFIX virus was strongly restricted to the liver with virtually no detectable virion accumulation in any other organ (Figure 1). This demonstrates a strong hepatic targeting of AAV8 in a dose dependent manner and was evident at all doses tested (Figure 1). Although the liver is clearly the predominant site for virion sequestration, the pattern in rats is modestly different to both mouse and non-human primates [11-13]. In mice significant levels of virion accumulation were detected in kidney and lungs in addition to liver [13]. In monkeys the majority was localised to liver but splenic accumulation was also observed [13].

**Figure 2:**
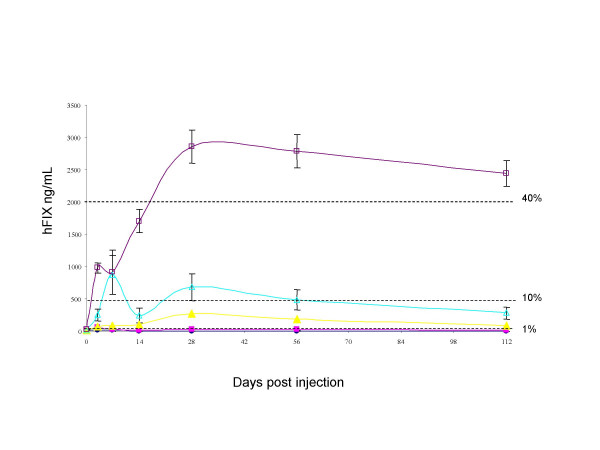
**Determination of circulating human factor IX levels in blood plasma of AAV8hFIX infused rats**. Blood plasma samples from scAAV8-LP1-hFIX infused rats at (black sqaure) 1 × 10^10^vg/rat, (black triangle) 5 × 10^10^vg/rat, (open triangle) 1 × 10^11^vg/rat, (open square) 5 × 10^11^vg/rat or (black circle) saline were assayed by ELISA to measure circulating hFIX activity. All values represent average hFIX levels with standard error of mean. Percentages refer to the percentage of normal circulating FIX levels.

To determine the level of transgene expression circulating in the blood following scAAV8 delivery in rats we performed enzyme-linked immunosorbent assay specific for hFIX on plasma samples taken from rats over the course of the experiment (4 months). Human FIX was detectable in the blood within 3 days and continued to rise until reaching peak levels at 4 weeks, with values of approximately 2.9 ± 0.26 μg/ml, 0.7 ± 0.2 μg/ml, 0.3 ± 0.06 μg/ml and 0.03 ± 0.01 μg/ml with administration of 5 × 10^11^, 1 × 10^11^, 5 × 10^10^, and 1 × 10^10 ^vector genomes/rat, respectively (Figure 2). The plasma concentration of hFIX achieved from a single intravenous injection of 5 × 10^11 ^vg/rat scAAV8-LP1-hFIX produced over 40% physiologic level in just 4 weeks. Infusion of lower doses of 1 × 10^11 ^and 5 × 10^10^vg/rat scAAV8-LP1-hFIX produced moderate increases in circulating transgene expression. The difference in transgene expression between vector doses was highly significant (P < 0.0001, by one-way ANOVA) demonstrating a dose response throughout the dosing regimens employed.

**Figure 3:**
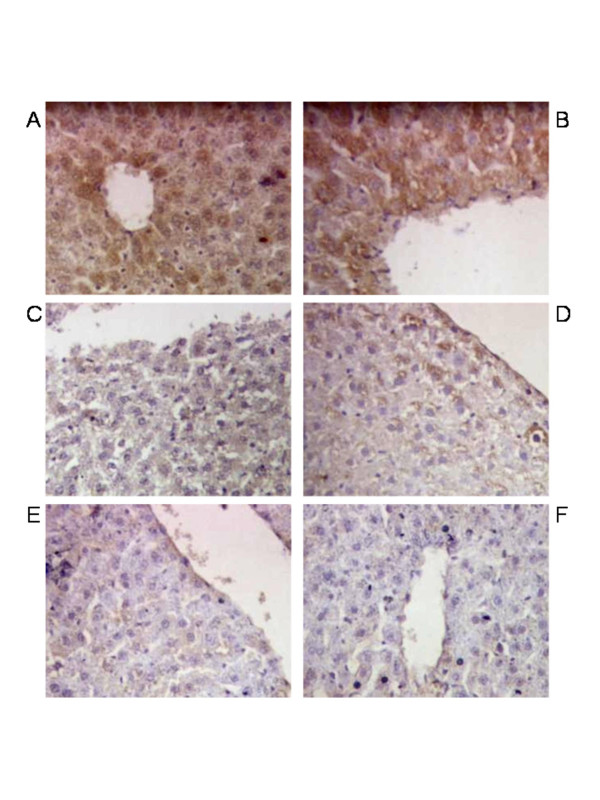
**Histological analysis of human FIX expression in livers of AAV8-LP1-hFIX infused rats**. Representative liver sections of immunohistochemical staining for hFIX 4 months after gene transfer in rats infused with scAAV8-LP1-hFIX at (A) 5 × 10^11^vg/rat, (B) 1 × 10^11^vg/rat, (C) 5 × 10^10^vg/rat, (D) 1 × 10^10^vg/rat or (E) saline. Paraffin liver sections were stained for human FIX expression using goat anti-human FIX conjugated to horse-radish peroxidase and goat IgG was used as the negative control (F). Magnification is × 40.

Immunohistochemical staining of the liver after intravenous administration of scAAV8-LP1-hFIX at doses of 1 × 10^11 ^and 5 × 10^11 ^vg/rat showed expression of hFIX in the hepatocytes at 16 weeks that was substantially higher than hFIX expression in the hepatocytes from rats infused with the lower doses of 1 × 10^10 ^and 5 × 10^10 ^vg/rat (Figure 3). A clear gradient in expression is seen with the most efficiently transduced cells around the portal tract (Figure 3), consistent with observations in mice [13]. No expression was seen in the heart of any of the animals by hFIX staining (data not shown), again confirming the hepatic selectivity of this vector construct.

## Conclusion

In summary, we have demonstrated herein that AAV8 can be used to mediate long term stable expression of transgene at therapeutic levels in the rat. This study confirms those conducted in other species relating to the strong hepatic tropism of AAV2/8 following peripheral vein injection and underscores the utility of this virus for gene therapy applications targeted to the liver.

## Competing interests

The author(s) declare that they have no competing interests.

## Authors' contributions

TG performed experiments. LW performed experiments. JM performed the ELISAs and prepared the virus. AN participated in the design of the study and provided virus. AB designed the study and drafted the paper. All authors read and approved the final manuscript
